# Short-Term Efficacy of 2% Topical Diltiazem in Fissure in Ano: A Prospective Observational Study From a Tertiary Care Centre in a Sub-Himalayan Region in India

**DOI:** 10.7759/cureus.90865

**Published:** 2025-08-24

**Authors:** Saurabh Pangtey, Praveen Kumar, Divyanshu Ghildiyal

**Affiliations:** 1 General Surgery, Himalayan Institute of Medical Sciences, Dehradun, IND

**Keywords:** anal fissure, chemical sphincterotomy, pain management, topical diltiazem, visual analog scale

## Abstract

Introduction: An anal fissure is a common anorectal condition characterised by a linear tear in the lower anal canal, causing severe pain and rectal bleeding. Topical chemical sphincterotomy using agents such as diltiazem has become a popular non-surgical treatment option.

Methods: This prospective observational study was carried out over 12 months at the Department of Surgery, Himalayan Institute of Medical Sciences, Dehradun, India. Six hundred patients aged 18-60 years with acute, acute-on-chronic, or chronic anal fissure and no previous treatment history were enrolled. Patients received 2% topical diltiazem applied locally twice daily for eight weeks. The amount of ointment applied was equivalent to the size of a pea; the patients were compliant throughout the study. Pain was evaluated using the visual analogue scale (VAS) at baseline, and after the first, second, and third weeks, as well as at three months. Data on side effects, the need for surgery, and fissure recurrence were also documented.

Results: The mean age was 34.17 ± 8.90 years, with a slight female predominance (53.3%). Pain scores decreased significantly over three months (p<0.001), with 75% of patients pain-free at three months. Mild perianal redness and itching were reported in 4.1% during the first week, with no adverse effects thereafter. Surgical intervention was required in 4% of cases by three months, and fissure recurrence occurred in 5%.

Conclusion: Topical 2% diltiazem is an effective, well-tolerated, and safe treatment for anal fissures, providing significant symptomatic relief and good healing potential with minimal side effects.

## Introduction

A painful linear tear in the distal anal canal that extends from just below the dentate line to the anal margin is known as an anal fissure (sometimes referred to as a fissure in ano) [1]. Although it can affect people of all ages, young, healthy adults are the most commonly affected, with the incidence being similar across genders. Because highly sensitive spinal neurons supply the area, anal fissures often present with pain, bright red bleeding per rectum, mucous discharge, and constipation. The most common location is the midline, with 90% of fissures occurring posteriorly and 10% anteriorly.

Based on their form and chronology, fissures can be classified as either acute or chronic. If anal fissures are superficial, have clearly defined edges, and have existed for less than six weeks, they are considered acute. Most acute anal fissures heal either spontaneously or with conservative medical treatment. In contrast, chronic anal fissures (CAF) last longer than six to eight weeks and display various morphological features, such as ulcers with keratinous edges, hypertrophied anal papillae, exposed internal anal sphincter smooth-muscle fibres, and a sentinel tag at the external apex. CAF often requires medical intervention, such as medication or surgery [2,3].

Anal fissures have an unclear cause. Previously, it was believed that straining during defecation or severe constipation was the cause of anal fissure [4]. However, according to recent data, anal fissures are associated with increased internal anal sphincter tone and spasm. Therefore, it is hypothesised that elevated resting anal pressure is the leading cause of CAF [5]. This pressure reduces anodermal blood flow by compressing the blood vessels passing through the sphincter, which ultimately results in ischaemic ulceration of the anal mucosa.

Risk factors for anal fissure include low-fibre diets and prior anal surgery, where scarring can cause anal canal constriction, increasing susceptibility to injury from hard stools. Fissures are most commonly located in the posterior midline (99% in men, 90% in women). Anterior fissures are more frequent in women, often linked to childbirth trauma and weaker anterior anal canal support due to the adjacent vagina [6]. Other causes include anal intercourse, Crohn’s disease, sexually transmitted infections (syphilis, herpes), lymphoma, leukaemia, basal and squamous cell carcinoma, tuberculosis, and systemic diseases such as AIDS [6].

Symptoms of an anal fissure include pain during defecation and per rectal bleeding. It is often triggered by stiff or painful bowel movements, causing a tear in the anal canal. The underlying mechanism involves spasm of the internal anal sphincter, leading to reduced blood flow and delayed healing. Anal fissures are classified as primary (without an identifiable cause) or secondary (with an underlying condition) [7]. Secondary causes include constipation, childbirth, inflammatory bowel disease (e.g., Crohn’s disease), sexually transmitted or skin infections, dermatological conditions such as psoriasis, opioid use, chemotherapy, anal trauma (including surgery or anal intercourse), and bowel cancer [7].

Medical management of fissure in ano, introduced in 1993, involves chemical sphincterotomy using agents that relax the internal anal sphincter. These include botulinum toxin, isosorbide dinitrate, glyceryl trinitrate (GTN), and calcium channel blockers such as nifedipine and diltiazem. The aim is to break the cycle of pain, spasm, and ischaemia that causes fissure formation. Topical GTN (0.2%), applied two to three times daily for six to eight weeks, helps healing in up to two-thirds of patients by relaxing the sphincter and enhancing local blood flow [8]. However, its use is limited by side effects such as headache, orthostatic hypotension, syncopal episodes, and tachyphylaxis [8].

Later, it was also demonstrated that cytoplasmic calcium is a crucial factor in smooth muscle contraction. In 1999, Carapeti et al. used a calcium channel blocker, namely, diltiazem, and found that when applied locally, 2% diltiazem gel reduced the mean resting pressure. The onset of action is quick and is long lasting as compared to other agents. Additionally, unlike topical nitrates for treating anal fissures, it potentially causes fewer side effects, such as headaches. The need for lateral sphincterotomy can be avoided in up to 70% of cases [9]. This study aims to evaluate the short-term efficacy and safety of 2% topical diltiazem in reducing pain intensity, promoting fissure healing, and monitoring recurrence and adverse events in patients with chronic anal fissure, through a prospective observational design.

## Materials and methods

Study design and setting

A prospective observational follow-up study was conducted over 12 months in the Department of Surgery at Himalayan Institute of Medical Sciences, SRHU, Jolly Grant, Dehradun, India. The study was approved by the Institutional Ethics Committee on 08/09/2023, bearing letter number SRHU/HIMS/ETHICS/2025/189. Informed written consent was obtained from all participants prior to enrolment.

Sample size and sampling: The sample size was calculated using the formula:

n = (Z²(1-α/2) p(1-p)) / d²

where Z = 1.96 (for 95% confidence), p = 0.178 (proportion of anal fissure), and d = 10%. The calculated sample size was 570, rounded up to 600 for convenience. Consecutive sampling was employed, enrolling all eligible consenting patients during the study period.

Inclusion and exclusion criteria

Patients of either gender aged between 18 and 60 years, presenting with acute, acute-on-chronic, or chronic anal fissure and with no prior treatment history for the condition, were included in the study. Patients who refused to give consent or those with a history of previous treatment for anal fissure were excluded. Additionally, patients with chronic fissure in ano associated with complicating factors, those requiring anal surgery for concurrent anorectal diseases (e.g., haemorrhoids, abscesses, or fistulas), and pregnant women were not considered eligible for participation in the study. All patients included in the study were given 2% diltiazem ointment to be applied twice daily. To standardise the amount, patients were advised to apply the ointment with a fingertip with an amount equal to the size of a pea.

Study tools

A case recording form was employed to document the demographic details and clinical data of all patients enrolled in the study. Pain severity was evaluated using the visual analog scale (VAS), a validated instrument comprising a 10 cm horizontal line with endpoints representing ‘no pain’ at 0 and ‘worst imaginable pain’ at 10. Patients were asked to mark a point along the line that best reflected their pain intensity at the time of assessment. The score was then measured in centimetres from the ‘no pain’ end to the patient’s mark. For analysis purposes, VAS scores were categorised into three groups: scores ≤3 were regarded as mild pain, scores between 3 and 7 were classified as moderate pain, and scores ≥7 were recorded as severe pain [10].

Data management and statistical analysis

Data were entered and analyzed using Microsoft Excel (Microsoft® Corp., Redmond, WA). Categorical variables were presented as numbers and percentages, and continuous variables as mean ± standard deviation (SD). The McNemar test was employed for categorical data. A p-value < 0.05 was regarded as statistically significant.

## Results

The study enrolled 600 patients with a mean age of 34.17 ± 8.90 years. The majority fell into the 31-40 years age bracket, depicting an early middle-aged population as the most commonly affected group. Interestingly, the study observed a slightly higher proportion of females (53.3%) than males (46.7%), suggesting a potential gender-based predisposition or differences in healthcare-seeking behaviour among women.

Occupational background further enriched the demographics: 30% were housewives, followed by farmers (20%) and teachers (16.7%). This varied patient profile underscores the broad social impact of the condition studied (Table 1).

**Table 1:** Sociodemographic determinants of the enrolled patients.

Variable	Domain	Number	Percentage
Age group	Mean age	34.17 ± 8.90 years	
	<21 Years	30	5.0
	21-30 Years	200	33.3
	31-40 Years	220	36.7
	41-50 years	140	23.3
	>50 years	10	1.7
Gender	Male	280	46.7
	Female	320	53.3
Occupation	Housewife	180	30.0
	Farmer	120	20.0
	Teacher	100	16.7
	Cook	90	15.0
	Serviceman	80	13.3
	Student	30	5.0

The level of pain was found to decrease significantly over a period of three months. In the first week, all patients (100%) experienced severe pain. By the second week, 55% of patients had moderate pain, and 45% had severe pain. In the third week, 78.3% of patients experienced mild pain, 16.7% had moderate pain, and 5% had severe pain. At the three-month follow-up, 75% of patients had no pain, 3.3% experienced moderate pain, and 21.7% had severe pain (Tables 2-3).

**Table 2:** Pain assessment of study participants over a three-month follow-up.

Week	Pain level	Number	Percentage		
1^st^ week	No pain	0	0		
	Mild pain	0	0		
	Moderate pain	0	0		
	Severe pain	600	100		
2^nd^ week	No pain	0	0		
	Mild pain	0	0		
	Moderate pain	330	55		
	Severe pain	270	45		
3^rd^ week	No pain	0	0		
	Mild pain	470	78.3		
	Moderate pain	100	16.7		
	Severe pain	30	5		
3^rd^ month	No pain	450	75		
	Mild pain	0	0		
	Moderate pain	20	3.3		
	Severe pain	130	21.7		

**Table 3:** McNemar test applied.

Time Interval	Improved (b)	Not Improved (c)	McNemar Test (χ²)	p-value	Result
Week 1 → Week 2	330	270	6.00	0.014	Significant
Week 2 → Week 3	470	130	192.67	< 0.0001	Highly Significant
Week 3 → Month 3	450	150	150.00	< 0.0001	Highly Significant

During the first week, 4.1% of patients experienced itching along with redness. No side effects were observed at the second week, third week, or at the three-month follow-up. Surgery was required at a significantly higher rate at three months (5%) compared to the second week (2%). Recurrence of fissure was observed in 5% of patients at the three-month follow-up (Tables 4-5).

**Table 4:** Assessment of side effects, surgery requirements, and recurrence in patients over a three-month period.

Variable	Domain	Number	Percentage
Side effects (Itching and redness)	Redness	25	4.1
	1^st^ week	0	0
	2^nd^ week	0	0
	3^rd^ week	0	0
	3^rd^ month	0	0
Surgery required	1^st^ week	0	0
	2^nd^ week	12	2
	3^rd^ week	0	0
	3^rd^ month	25	4
Recurrence	1^st^ week	0	0
	2^nd^ week	0	0
	3^rd^ week	0	0
	3^rd^ month	30	5

**Table 5:** McNemar test applied.

Variable	Timepoints Compared	Improved (b)	Worsened (c)	McNemar Test (χ²)	p-value	Result
Surgery Required	2nd Week vs 3rd Month	2	15	9.94	0.0016	Significant
Recurrence	3rd Week vs 3rd Month	0	30	30.00	< 0.00001	Highly Significant

## Discussion

CAF can be effectively treated with pharmacological or surgical sphincterotomy. Although lateral internal sphincterotomy offers healing rates of up to 95%, it poses a significant risk of incontinence. As a result, chemical sphincterotomy has become more popular, with agents such as oral and topical calcium channel blockers (CCBs) reducing mean resting anal pressure by 21-28% in healthy volunteers. While oral diltiazem has demonstrated effectiveness in healing fissures, side effects such as postural dizziness, facial flushing, and mild headaches have been reported. Topical diltiazem, which has minimal systemic absorption, provides a better side-effect profile [9]. The present study assesses the effectiveness of topical diltiazem in alleviating pain, reducing spasm, and promoting healing in patients with fissure in ano.

In this study, the mean patient age was 34.17 ± 8.90 years, with most patients aged between 31 and 40 years (36.7%). This aligns with other research, such as that by Bansal et al. [1], Griffin et al. [11], Jonas et al. [12], and Vaithianathan et al. (35.76 years) [13]. Similarly, Swarnkar et al. reported an average age of 38.96 ± 13.93 years [14], Tavakoli-Dastjerdi et al. found a mean age of 39.61 ± 10.09 years [15], and Venkatesh et al. observed ages of 38 years in the diltiazem group and 36 years in the GTN group [16].

A female predominance of 53.3% was observed, aligning with Jonas et al. [12] and Swarnkar et al. [14]. However, other studies, such as Bansal et al. [1] and Griffin et al. [14], reported male-dominant or nearly equal distributions. Kulkarni et al. reported a higher female preponderance of 72% [17], whereas Sharma et al. observed mixed gender ratios in their oral and topical diltiazem groups [18].

Pain, the hallmark symptom of CAF, showed significant improvement in this study. At baseline, all patients reported severe pain, which decreased progressively over the study period. By the three-month follow-up, 75% of patients were pain-free. This aligns with Bansal et al., who observed perceptible pain relief by six weeks [1], Griffin et al., who noted significant improvements by eight weeks [11], and Jonas et al., who reported a reduction in symptoms after two weeks [12]. Ala et al. also noted greater initial pain reduction with diltiazem compared to GTN, although the results equalised by the eighth week [19]. Similar progressive improvement was documented by Swarnkar et al. [14] and Venkatesh et al. [16], with significant pain relief achieved by four weeks in both the GTN and diltiazem groups [16].

Adverse effects were minimal in this study, with 13.3% experiencing perianal redness and 3.3% reporting itching in the first week, which resolved afterwards. Similar findings were observed in Bansal et al. (8% headaches) [1], Knight et al. (4 cases of perianal dermatitis, 1 headache) [20], and Griffin et al. [11], where a small number of patients experienced a localized rash and perianal itching. Jonas et al. noted adverse effects mostly with oral diltiazem [12], and Ala et al. found a significantly better side-effect profile for diltiazem compared to GTN, which caused headaches in all treated patients [19]. Swarnkar et al. reported mild headaches and perianal itching in a minority [14], while Venkatesh et al. [16] documented headaches in 14% and pruritus in 6% of cases treated with diltiazem. Momayez Sanat et al. observed minimal side effects, mainly dizziness and headaches (2.44%) [21].

The need for surgical intervention in this study rose from 2% at two weeks to 4% at three months, emphasising cases unresponsive to medical therapy. Knight et al. observed similar trends, where patients unhealed after eight weeks of diltiazem either switched to GTN or underwent sphincterotomy [20].

Recurrence at three months was observed in 5% of patients in this study. This aligns with Knight et al., who recorded seven recurrences over 32 weeks [20], and Griffin et al., who documented two relapses during follow-up [11]. Jonas et al. reported one recurrence per group at four and six months, respectively [12], while Tavakoli-Dastjerdi et al. observed a lower recurrence rate in the intervention group [15]. Momayez Sanat et al. noted recurrence rates of 21.4% for diltiazem at six months, which is comparable to this study’s three-month results [21]. Sharma et al. documented recurrences in four patients by three months, evenly distributed between oral and topical diltiazem groups [18].

A notable limitation of this study was the absence of a control group, which hampers definitive comparison of outcomes with standard therapies. Furthermore, variability in ointment quantity, application site, and patient compliance may have affected the results. Although diltiazem effectively reduces resting anal pressure for up to five hours in healthy individuals [9], its exact duration of action in fissure patients remains uncertain. Adjusting the dose, concentration, and administration schedule based on pharmacodynamic effects could potentially improve outcomes.

Given its comparable healing rates to GTN without severe side effects, topical 2% diltiazem provides a promising first-line treatment for CAF. Several studies support its role in managing fissures unresponsive to GTN [22]. Further long-term, controlled trials are necessary to assess its sustained efficacy, recurrence rates, and effectiveness in GTN-resistant cases.

A key limitation of this study is the absence of a control group, making it hard to compare topical diltiazem with other standard treatments or a placebo. Because the study was neither randomized nor blinded, there is a risk that unconscious bias - both in selecting patients and in how outcomes such as pain were reported - may have affected the results. Pain being subjective, responses could be unintentionally influenced without blinding. Additionally, as an observational study, it lacks the scientific rigor of a randomized controlled trial.

The follow-up period of only three months may not be sufficient to assess the long-term effectiveness, side effects, or recurrence rates. There was also an inconsistency in ointment application, as patient compliance and the amount used were not monitored, which could impact outcomes.

Conducted at a single hospital in the sub-Himalayan region, the study’s findings may not be generalizable to other populations. It also did not evaluate quality of life or functional outcomes, and the criteria for surgical referral were not clearly defined. Finally, the report does not specify whether any participants dropped out, which could affect the validity of the conclusions.

## Conclusions

The present study was a prospective study that assessed the efficacy of topical diltiazem in relieving pain and spasm in patients with fissure in ano and to evaluate the effectiveness of topical diltiazem in the healing of fissures. The highest incidence of the disease was found in the fourth decade of life, with a predominance of females. With the application of 2% topical diltiazem, the level of pain was found to decrease significantly over a period of three months. Itching and redness were observed as side effects in a few cases. Recurrence of fissure was observed in 5% of patients at three months of follow-up. In conclusion, it has been found that topical diltiazem effectively lessens symptoms of anal fissure. Anal fissures can be effectively treated with topical diltiazem, which has a good success rate. Thus, topical diltiazem is an effective approach with a good safety profile for alleviating anal fissure pain and promoting healing.

This study lacked a control group and randomisation, which limits the ability to establish causal relationships. The observational design and absence of blinding increase the risk of bias. Follow-up was restricted to three months, preventing assessment of long-term outcomes. The single-centre setting may limit the generalisability of results. No measures of quality of life or functional outcomes were evaluated. Furthermore, criteria for surgical referral were not standardised, and the study did not report on dropouts or lost to follow-up, which could introduce attrition bias and compromise the validity of the findings. This prospective observational study indicates that topical diltiazem may offer symptomatic relief in a majority of chronic anal fissure patients in the short term. However, due to the absence of a comparator group and short follow-up, definitive conclusions regarding its efficacy and long-term outcomes require validation through controlled trials.
